# Multitracer PET/CT with [^18^F]Fluorodeoxiglucose and [^18^F]Fluorocholine in the Initial Staging of Multiple Myeloma Patients Applying the IMPeTus Criteria: A Pilot Study

**DOI:** 10.3390/diagnostics13091570

**Published:** 2023-04-27

**Authors:** Puy Garrastachu Zumarán, Irene García Megías, María Mangas Losada, Alejandro Mendoza Melero, Amós Villanueva Torres, Xavier Boulvard Chollet, Leonardo Romero Robles, Prisma Montserrat Hernández Pérez, Rafael Ramírez Lasanta, Roberto C. Delgado Bolton

**Affiliations:** 1 Department of Diagnostic Imaging (Radiology) and Nuclear Medicine, University Hospital San Pedro, 26006 Logroño, Spain; 2 Centre for Biomedical Research of La Rioja (CIBIR), Fundación Rioja Salud, 26006 Logroño, Spain; 3 Department of Haematology, University Hospital San Pedro, 26006 Logroño, Spain

**Keywords:** PET/CT, FDG, FCH, multiple myeloma, IMPeTus criteria, staging, bone marrow, focal lesions, extra-medullary disease, para-medullary disease

## Abstract

Initial staging of patients diagnosed with multiple myeloma (MM) can lead to negative results using conventional diagnostic imaging workup, including [^18^F]Fluorodesoxiglucose ([^18^F]FDG) PET/CT. The aim of this prospective pilot study was to evaluate the diagnostic efficacy of [^18^F]Fluorocholine ([^18^F]FCH) PET/CT in the initial staging of MM patients who were candidates for autologous bone marrow transplant. **Materials and Methods**: The inclusion criteria of our study were: (a) patients diagnosed with MM; (b) candidates for autologous bone marrow transplant (AT); and (c) studied with [^18^F]FCH PET/CT and [^18^F]FDG PET/CT for initial staging less than 4 weeks apart. Imaging analysis included the presence of: bone marrow infiltration, focal bone lesions, and para-medullary or extra-medullary disease, according to the proposed IMPeTus criteria. The analysis was performed per lesion, per patient, and per location. **Results**: The study population included ten patients. Globally, [^18^F]FCH PET/CT showed bone marrow uptake in all the patients and visualised 16 more focal lesions than [^18^F]FDG PET/CT. One patient presented a plasmacytoma, detected by both tracers. Extra-medullary and para-medullary disease was identified with different degrees of uptake by both tracers. In summary, [^18^F]FCH PET seemed to be superior to [^18^F]FDG PET/CT in detecting focal bone lesions. SUVmax values were slightly higher in [^18^F]FCH PET/CT than in [^18^F]FDG PET/CT. **Conclusions**: Taking into account the small study population, according to our results, [^18^F]FCH PET/CT could be a useful tool for staging MM patients.

## 1. Introduction

Multiple myeloma (MM) is a haematological disease characterised by the uncontrolled clonal proliferation of tumoral plasma cells (PC) in the bone marrow (BM). It comes under a group of disorders that receive the name of monoclonal gammopathies [1,2].

MM represents around 10% of haematological diseases [3], being the second most common process just preceded by non-Hodgkin Lymphoma. The mean age of diagnosis is around 69 years, with an estimated number of new cases in 2022 of 34,400 [4]. According to the latest Spanish guide for MM [5], each year about 2500–3000 new cases are diagnosed in our country. Of these, only 36% are candidates for an autologous bone marrow transplant. The criteria for considering patients candidates for an autologous bone marrow transplant are: age less than 65 years, without medical contraindications. Between 65 and 70 years old, patients have to be evaluated individually. An absolute contraindication is hepatic cirrhosis, even if clinically compensated. Relative contraindications are the presence of non-controlled infections, chronic liver disease (non-cirrhosis), left ventricular ejection fraction below 40%, diffusing capacity of the lung for carbon monoxide (DLCO) below 40%, and creatinine extraction fraction below 30 mL/min [5].

Notwithstanding the advances in its treatment, leading to a survival rate that has doubled in the last decade (with a 5-year relative survival of 57.9% nowadays) [4], it remains an incurable disease. 

Currently, the accepted definition of MM includes a clonal proliferation of more than 10% of the plasma cells in bone marrow, accompanied by one or more myeloma-defining events that may be either the presentation of organic dysfunction or the presence of biomarkers of malignancy. Within the associated organic dysfunction, there can be: hypercalcemia, renal failure, anaemia, or bone lesions, as summarised by the acronym “CRAB criteria”. On the other hand, malignancy biomarkers include a percentage of clonal bone marrow plasma cells greater than or equal to 60%, a ratio of free light chain equal to or greater than 100, and the detection of more than one focal lesion on magnetic resonance imaging (MRI) [1].

The combination of morphological and functional images offered by [^18^F] Fluorodesoxiglucose ([^18^F]FDG) PET/CT plays a central role in MM in both initial staging and follow-up [6]. If we focus on staging, it shows high rates of sensitivity and specificity (90% and 70–90%, respectively) [7]. Along with MRI, performing [^18^F]FDG PET/CT is highly recommended by the International Myeloma Working Group for both baseline staging and the evaluation of response to treatment in the follow-up [8]. Both techniques have demonstrated a good concordance in almost every location [9].

[^18^F]FDG PET/CT provides information about glycolytic activity, which is increased in almost all tumoral malignancies. Thus, [^18^F]FDG PET/CT has been considered one of the cornerstones in the management of MM. While it is a well-established imaging technique, there was a lack of established criteria for categorising the degree of disease burden in MM. In this scenario, an Italian group of nuclear medicine experts, haematologists, and medical physicists defined new visual descriptive criteria based on the Deauville score (Italian Myeloma criteria for Pet Use: IMPeTUs) to standardise [^18^F]FDG PET/CT evaluation in MM patients [10] and proved them to be highly reproducible.

However, [^18^F]FDG is not exempt from limitations. It is well-known that it presents negative results when staging some patients with MM and diffuse infiltration of bone marrow or a low expression of hexokinase-2 (HK-2), with false-negative results in up to 10% of cases [11].

With this premise, there is a clear need to search for other radiotracers to improve diagnostic accuracy. Among all the new radiopharmaceuticals under study, we explored the possible role of [^18^F]Fluorocholine ([^18^F]FCH) PET/CT, which has been proved to be superior to [^18^F]FDG PET/CT in some scenarios.

Choline, as a precursor of phospholipids, can be used as an indicator of membrane synthesis, as there is evidence of its increased levels in MM microenvironments. The enhanced choline-kinase activity steers to an augmented choline uptake. The usefulness of choline in MM was first described by Ambrosini et al. [12] in an incidental finding in which they observed choline uptake by a solitary plasmacytoma in a patient with monoclonal gammapathy of undetermined significance (MGUS) undergoing [^11^C]-Choline-PET/CT for suspected relapse of primary prostatic cancer.

Whereas the superiority of [^18^F]FCH PET/CT has been demonstrated in patients already diagnosed with MM presenting suspicion of relapse or progression, there is a lack of evidence regarding its role in the initial staging of these patients [13]. Additionally, the availability of larger baseline studies using choline tracers would allow the assessment of their prognostic significance, already demonstrated for [^18^F]FDG PET/CT [14].

The aim of this prospective pilot study was to compare lesion detection with [^18^F]FCH PET/CT versus [^18^F]FDG PET/CT in the initial staging of MM patients who were candidates for autologous bone marrow transplant, using the IMPeTUs criteria for the evaluation of both PET/CT studies. Very strict inclusion criteria, established in conjunction with the haematologists, were defined.

## 2. Materials and Methods

### 2.1. Patient Selection

The inclusion criteria of this prospective pilot study were: (a) patients diagnosed with MM; (b) who were candidates for autologous bone marrow transplant (see above); and (c) studied with [^18^F]FCH PET/CT and [^18^F]FDG PET/CT for initial staging, with both scans performed in a time interval of less than four weeks.

Ten patients (4 male, 6 female; age range 43–73 years) recently diagnosed with MM and candidates for an autologous bone marrow transplant were enrolled. Written informed consent was obtained before undergoing both [^18^F]FCH PET/CT and [^18^F]FDG PET/CT, which were performed with an interval that ranged between 1 and 28 days, with a median of 4 days (Table 1). The order of the studies was not considered relevant.

The first PET/CT was performed in November 2018 and the last one in April 2019.

All patients had a “de novo” diagnosis of MM and were candidates for autologous bone marrow transplant. One patient with chronic myeloid leukaemia was included in the study. The remaining participants had no prior or concurrent tumoral pathology during the study.

Informed consent was obtained from all patients involved in the study. Ethical approval was sought and received from an independent ethical committee (Comité de Ética de Investigación con medicamentos de La Rioja; Ref. CEImLAR-PI-270).

### 2.2. [^18^F]FDG PET/CT and [^18^F]FCH PET/CT Procedures

Both [^18^F]FDG PET/CT and [^18^F]FCH PET/CT were performed using the standard protocol for each technique [15,16]. Preparation prior to image acquisition included fasting for at least 4 h and proper hydration. 

[^18^F]FCH was injected following a quadratic relation between [^18^F]FCH dose and the patient’s body mass. Whole-body PET/CT acquisition was performed 60 min after injection. Choline tracers have physiological uptake by the liver, spleen, pancreas, salivary and lachrymal glands, and urinary tract (due to renal excretion). Less uptake tracer could be seen in bone marrow and intestine [17].

[^18^F]FDG was injected following a quadratic relation between [^18^F]FDG dose and the patient’s body mass [18]. Whole-body PET/CT acquisition was performed 60 min after injection. [^18^F]FDG has physiological uptake by the brain, myocardium, kidneys, urinary tract, liver, and spleen.

Imaging was performed in an integrated PET/CT scanner (Siemens Biograph 6 Hi-Rez). Attenuation correction was performed with a low-dose spiral CT-based method (120 kV, 80 mA) and PET emission data were acquired in 2-dimensional mode. PET data were reconstructed using a method of ordered-subsets (8 subsets, 4 iterations, and Gaussian filtering).

### 2.3. [^18^F]FDG PET/CT and [^18^F]FCH PET/CT Image Analysis

Each PET/CT study was evaluated using both visual (using axial, coronal, and sagittal views) and semiquantitative analyses by a nuclear medicine physician with expertise in [^18^F]FDG PET/CT and [^18^F]FCH PET/CT. 

To evaluate the diagnostic accuracy of both techniques, we performed a comparison per patient, per lesion, and per location. The parameter calculated was the detection rate.

The total number of lesions detected in each patient with [^18^F]FDG and [^18^F]FCH were calculated. SUVmax values were calculated in all the lesions detected with [^18^F]FDG PET/CT and [^18^F]FCH PET/CT. In patients with multiple lesions, the target or most intense lesions for each of the radiotracers, [^18^F]FDG and [^18^F]FCH, were compared.

As dictated by IMPeTus criteria, we analysed the metabolic activity of bone marrow, number and site of positive focal lesions, the number of lytic lesions, fractures, and the presence and location of extra-medullary and para-medullary disease. Extra-medullary disease can be divided in nodal and extranodal implication. As previously described, Deauville score is a 5-point scale used in daily practice in the assessment of lymphoma [19]. To establish a grade within the Deauville scale, different volumes of interest (VOIs) were set in the lesion of interest, liver parenchyma, and aorta in order to compare the maximum standardised uptake value (SUVmax).

Each uptake occurring in non-physiological sites was analysed to exclude non-tumoral lesions.

### 2.4. Reference Standard

The reference standard included medical imaging (including follow-up PET/CTs), clinical findings, and response to treatment.

### 2.5. Statistical Analysis

Quantitative values, such as age, were summarised with range and median (Table 1). No qualitative parameters were analysed.

To evaluate the diagnostic accuracy of [^18^F]FDG PET/CT and [^18^F]FCH PET/CT, the parameter calculated was the detection rate, performing a comparison per patient, per lesion, and per location, taking into account the four different components of MM including: focal bone lesions, bone marrow infiltration (differentiating between diffuse and patchy infiltration), and the presence of extra-medullary and para-medullary disease. 

## 3. Results

Ten patients (four male, six female; median age of 59.6 years at diagnosis, age range 43–73 years) with newly diagnosed MM and who were candidates for an autologous bone marrow transplant were included in our study (Table 1).

Table 2 presents the description of the location and main characteristics of the MM lesions detected with both PET/CT studies and a lesion-by-lesion comparison between [^18^F]FDG PET/CT and [^18^F]FCH PET/CT.

SUVmax values were calculated for [^18^F]FDG PET/CT and [^18^F]FCH PET/CT in all the lesions detected with either technique. Table 3 summarises the SUVmax values for [^18^F]FDG PET/CT and [^18^F]FCH PET/CT, as well as the total number of lesions detected in each patient with [^18^F]FDG and [^18^F]FCH. In patients with multiple lesions, the target or most intense lesions for each of the radiotracers, [^18^F]FDG and [^18^F]FCH, are compared and presented, including the Deauville score for each radiotracer.

To evaluate the extent of disease at staging we used the proposed IMPeTus criteria (Italian myeloma criteria for PET use).

### 3.1. Patient-Based Analysis

The analysis revealed that five of our patients (50%) with no suspicion of BM infiltration by [^18^F] FDG PET/CT showed pathological uptake in [^18^F]FCH PET/CT. The predominant pattern seen by [^18^F]FCH PET/CT was a diffuse uptake (90%).

Regarding the presence of focal lesions, two of our patients (20%) showed more axial foci in [^18^F]FCH PET/CT versus [^18^F]FDG PET/CT. In one of those patients, one of the axial lesions was finally categorised as degenerative.

**Figure 1:**
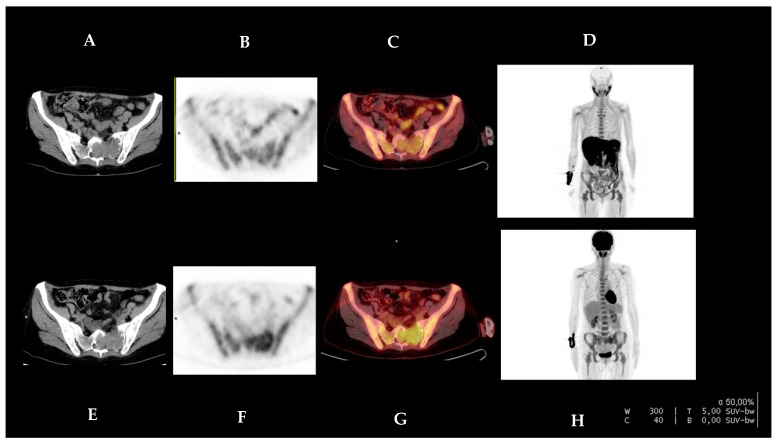
Sixty-seven year-old female (patient #3) presented with plasmacytoma in left sacrum. [^18^F]FCH PET/CT (upper row, (**A**–**D**)) and [^18^F]FDG PET/CT (lower row, (**E**–**H**)) are presented, corresponding to the CT, PET, PET/CT fusion, and MIP (maximum intensity projection). The images show higher uptake with [^18^F]FDG than with [^18^F]FCH (SUVmax 4.5 vs. 4.1, respectively) in the lesion in the left sacrum. The [^18^F]FCH PET/CT and [^18^F]FDG PET/CT images are presented with equal grey-scale intensity.

One patient presented with osseus plasmacytoma located in the sacrum. It was detected by both radiotracers, although the intensity of the uptake was higher with [^18^F]FDG than with [^18^F]FCH (4.5 vs. 4.1, respectively) (Figure 1).

**Figure 2:**
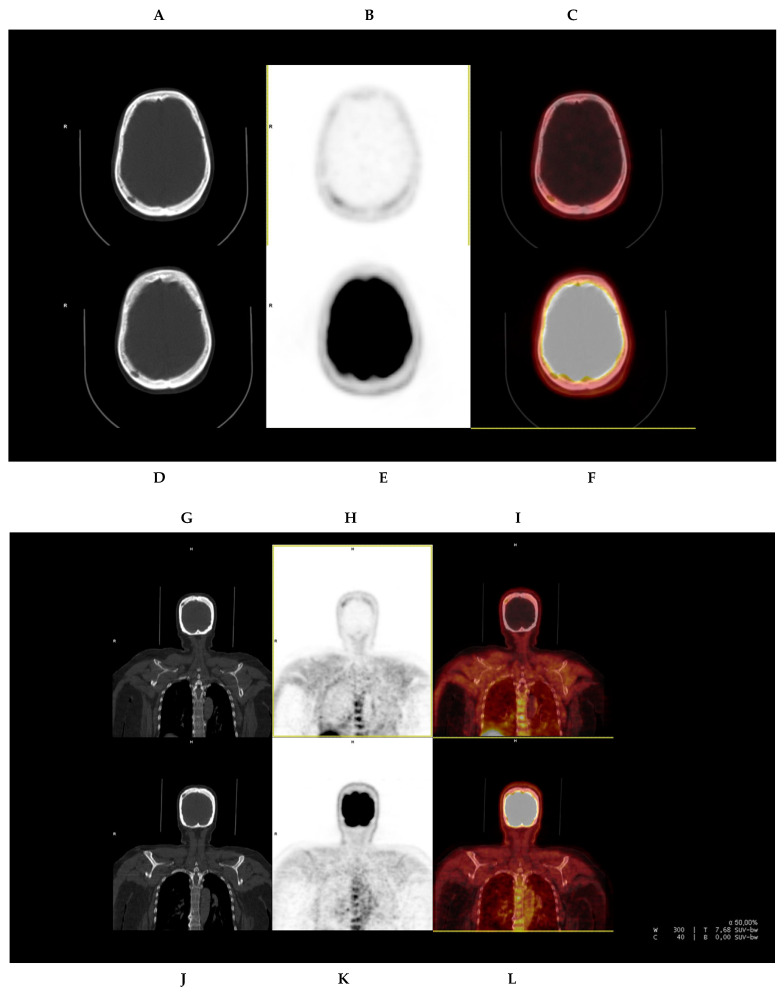
Fifty-nine year-old male (patient #7) presenting multiple lytic lesions located in the cranial calvarium. [^18^F]FCH PET/CT (upper row, (**A**–**C**,**G**–**I**)) and [^18^F]FDG PET/CT (lower row, (**D**–**F**,**J**–**L**)) are presented, corresponding to the axial (**A**–**F**) and coronal (**G**–**L**) projections of CT, PET, and PET/CT fusion, respectively. The images show [^18^F]FCH uptake in the lytic lesions (SUVmax 1.6), which had no [^18^F]FDG uptake. These differences are evident in both the axial and coronal projections. The [^18^F]FDG PET/CT and [^18^F]FCH PET/CT images are presented with equal grey-scale intensity.

One of the patients with no [^18^F]FDG-positive focal lesions in the cranial calvarium, but with evidence of a lytic lesion in CT, showed at least five [^18^F]FCH-positive focal uptakes in the cranial calvarium related to myelomatous involvement (Figure 2).

Of the nine patients included, two of them showed [^18^F]FDG-positive lesions in the axial skeleton that were not avid for [^18^F]FCH. These images were finally related to a rib fracture and an arthropathy, respectively. 

In three patients, we identified extra-medullar foci. One of them showed two foci, one in the vagina with an inflammatory-infectious origin and a second one in the liver, considered unspecific. The remaining patients showed one [^18^F]FDG-positive foci related to inflammatory parotid adenopathy and another [^18^F]FCH foci corresponding to a preauricular adenopathy, considered malignant.

**Figure 3:**
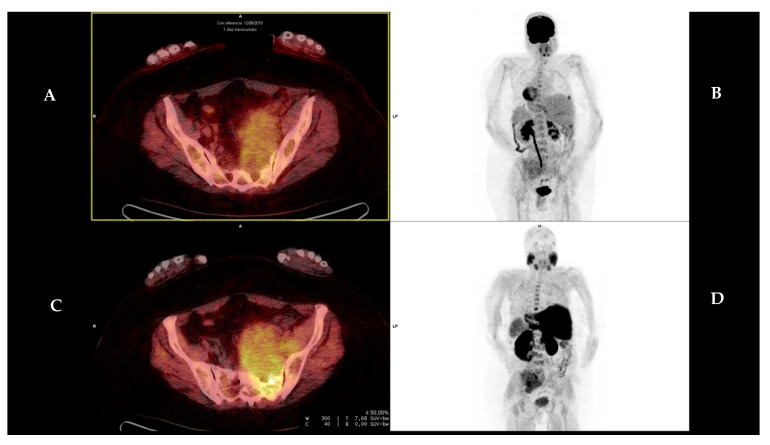
Fifty-nine year-old male (patient #7) presented para-medullary disease located in the left sacrum. [^18^F]FDG PET/CT (upper row, (**A**,**B**)) and [^18^F]FCH PET/CT (lower row, (**C**,**D**)) are presented, corresponding to the PET/CT fusion and MIP (maximum intensity projection). It shows higher uptake in [^18^F]FCH PET/CT than in [^18^F]FDG PET/CT, with SUVmax 8.9 vs. 6.9, respectively. The [^18^F]FCH PET/CT and [^18^F]FDG PET/CT images are presented with equal grey-scale intensity.

We identified para-medullary disease in two patients, with one lesion each. The first patient presented a lesion located in the left sacrum (Figure 3) and the second one presented a lesion in a rib, both of them showing higher uptake with [^18^F]FCH than with [^18^F]FDG. The lesion located in the left sacrum showed SUVmax 8.9 in [^18^F]FCH and 6.9 in [^18^F]FDG, while the one located in the rib showed SUvmax 4.0 versus 3.5 in [^18^F]FCH and [^18^F]FDG, respectively.

### 3.2. Lesion-Based Analysis

When evaluating bone marrow infiltration, both exams were concordant ([^18^F]FDG-positive and [^18^F]FCH-positive) in five patients (55.55%). The remaining four patients were only positive in [^18^F]FCH PET/CT, with predominant diffuse infiltration patterns (88.88%) versus a patchy infiltration pattern, only seen in one patient (11.11%).

In our study, [^18^F]FCH PET seems to be superior to [^18^F]FDG PET/CT in the determination of focal bone lesions. [^18^F]FCH PET/CT detected 16 more focal lesions than [^18^F]FDG PET/CT.

We identified one plasmacytoma with greater avidity for [^18^F]FDG than [^18^F]FCH (4.5 versus 4.1, respectively).

Cranial calvarium lesions were only identified by [^18^F]FCH PET/CT.

There were two bone lesions only positive in [^18^F]FDG PET/CT, both suggestive of corresponding to a rib fracture and degenerative changes, respectively.

Three [^18^F]FDG-positive foci in extra-medullary locations were not identified by [^18^F]FCH PET/CT. They corresponded to an inflammatory-infectious lesion in the vagina, an unspecific focus in the liver, and an inflammatory parotid adenopathy.

One [^18^F]FCH-positive foci related to a malignant preauricular adenopathy was not identified by [^18^F]FDG PET/CT.

Regarding para-medullary disease, we described two lesions. Both of them showed higher uptake by [^18^F]FCH.

## 4. Discussion

The development of new radiotracers in the field of nuclear medicine has improved the approach of MM, overcoming some limitations intrinsic to [^18^F]FDG. When analysing the results of this study regarding the infiltration of bone marrow, [^18^F]FCH PET/CT showed better sensitivity in comparison with [^18^F]FDG PET/CT. In clinical practice, along with [^18^F]FDG, MRI is the other imaging technique used in the management of MM, with a demonstrated superior diagnostic accuracy in the evaluation of bone marrow infiltration [20]. A systematic review and meta-analysis, published in 2012, compared 14 studies (including 395 patients) and determined that MRI was superior to [^18^F]FDG PET/CT in the evaluation of bone marrow infiltration, while [^18^F]FDG PET/CT could be useful in the assessment of extra-medullary disease [21]. The superiority of MRI in detecting bone marrow involvement is clear, while there is a lack of studies comparing [^18^F]FCH PET/CT versus MRI. The physiological [^18^F]FCH uptake in bone marrow, which could be confusing when evaluating infiltration, must be taken into consideration as a limitation of this radiotracer. In addition, as our patients did not undergo MRI at baseline, one of our limitations consisted of the inability to refuse [^18^F]FCH inferiority. It would be interesting to compare both MRI and [^18^F]FCH PET/CT in this context, as the role of [^18^F]FCH in diffuse bone marrow infiltration is not clear. In any case, a study comparing [^11^C]Choline and [^11^C]Methionine suggests a good correlation between diffuse infiltration and choline activity in bone marrow [22].

[^18^F]FCH PET/CT also demonstrated to be superior to [^18^F]FDG PET/CT in detecting focal lesions. [^18^F]FCH PET/CT showed more foci, both in the lesion-based analysis and in the patient-based one. A pilot study ruled out by Cassou-Mounat et al. in 21 patients with suspicion of relapse or progressive disease revealed up to 75–76% more lesions using [^18^F]FCH [23]. It is noteworthy that there were no patients presenting lesions that were [^18^F]FDG-positive and [^18^F]FCH-negative, while those lesions only detected in [^18^F]FCH PET/CT were mostly located in the cranial calvarium and torso. The superiority of [^18^F]FCH in evaluating cranial calvarium lesions is also proved in our study, in which one patient with no [^18^F]FDG-positive lesions in the cranial calvarium, but with evidence of lytic lesions in the CT, showed at least five [^18^F]FCH-avid lesions (Figure 2). These false-negative findings in [^18^F]FDG PET/CT are based on the high physiological uptake by the brain, which is one of the main limitations of [^18^F]FDG PET/CT. 

[^18^F]FCH PET/CT seems to also be superior to [^18^F]FDG PET/CT in the assessment of axial involvement, as in our study practically all the focal bone lesions missed in [^18^F]FDG scan were located in the column. This finding has also been described in previous studies [24].

In the literature, nearly 10% of newly diagnosed MM patients have a negative [^18^F]FDG PET/CT, while there is evidence of bone disease in MRI. These findings are related to a lower expression of HK-2 [11], although other studies suggest that other subjacent mechanisms should be involved due to patients with [^18^F]FDG-negative PET and average levels of HK-2 [25].

The utility of [^18^F]FDG PET/CT has also been compared with [^11^C]Fluorocholine ([^11^C]FCH)PET/CT in patients with multiple myeloma [26], demonstrating a higher sensitivity of [^11^C]FCH PET/CT, but also showing lesions [^18^F]FDG-positive and [^11^C]FCH-negative with no certain explanation to that discordant finding. The main disadvantage of [^11^C]Fluorocholine is its shorter half-life (20 min), which makes it necessary to have an onsite cyclotron for its production. These studies also showed higher median SUVmax for [^18^F]FCH PET/CT and [^11^C]FCH PET/CT. Although radiotracer uptake differences are not comparable, because glycolytic activity cannot be compared with phospholipid synthesis, our results are in contrast with the study just cited. Practically all our patients showed similar SUVmax, and some lesions even showed higher rates of uptake for [^18^F]FDG than for [^18^F]FCH. The mechanism underlying this variability is unclear, but higher uptake entails higher background contrast, which leads to a better visualisation and better inter-observer agreement. In fact, this variation in uptake rates is seen in our study, as one patient presenting with plasmacytoma in the left sacrum showed a higher SUVmax in [^18^F]FDG PET/CT compared with that in [^18^F]FCH PET/CT.

In our study, two osseous foci where positive in [^18^F]FDG-PET/CT but showed no avidity for [^18^F]FCH. These findings had a non-oncological aetiology in the end and corresponded to a rib fracture and a degenerative lesion, respectively. It is worth mentioning that the finding of a [^18^F]FDG-positive/[^18^F]FCH-negative lesion could be seen not only in lesions of mechanical aetiology, but also in aggressive dedifferentiated lesions [23].

Regarding rib lesions, it is important to highlight the physiological liver parenchyma uptake of choline tracers that can mask real MM affectation in adjacent ribs. The high activity in the liver can also hide extra-medullary disease (liver involvement), and it is also worth mentioning that some benign hepatic lesions can show avidity for choline radiotracers, as, for example, focal nodular hyperplasia [27]. One of our patients showed a [^18^F]FDG-positive foci in the liver that was unspecific and which was finally considered as non-tumoral.

Two additional extra-medullary foci with non-malignant significance and which were positive in [^18^F]FDG-PET/CT were detected in our study: one focus located in the vagina, with an inflammatory/infectious origin, and another one related to a parotid adenopathy, with an inflammatory origin.

It is known that extra-medullary disease entails worse prognosis with shorter progression-free survival and overall survival, counting as a poor prognostic marker in newly diagnosed and relapsed MM [28]. We identified tumoral extra-medullary disease in one of our patients presented as a [^18^F]FCH-positive preauricular adenopathy. During follow-up, this patient had a relapse of the disease after initial treatment.

Para-medullary disease was detected by both tracers in two patients, with higher uptake in [^18^F]FCH PET/CT than with [^18^F]FDG PET/CT. Some of the studies previously cited [23,26] revealed a variability in the [^18^F]FCH uptake with no superiority of [^18^F]FCH PET/CT over [^18^F]FDG PET/CT.

One of our objectives was to evaluate the application of the IMPeTUs criteria in the initial evaluation of MM when staging with [^18^F]FCH PET/CT. As the IMPeTUs criteria use the liver uptake as a reference, and given the fact that the liver presents a high [^18^F]FCH liver uptake, the categorisation using the Deauville criteria would not be applicable. In fact, almost all of our patients were categorised as Deauville three in [^18^F]FCH PET/CT (90%). Just one patient was categorised as Deauville two.

The main limitation of this study is the small study population. First of all, this is a pilot study with very strict inclusion criteria, established in conjunction with haematologists in order to evaluate patients with newly diagnosed MM who were candidates for an autologous bone marrow transplant. In this selected patient population, there is no conclusive evidence regarding the role of [^18^F]FCH PET/CT and its performance compared with that of [^18^F]FDG PET/CT. Second, an important drawback was the difficulty in finding patients that met these criteria. Third, after the inclusion of the first 10 cases, the number of lesions detected with either [^18^F]FDG PET/CT or [^18^F]FCH PET/CT was low, as we therefore decided to stop the study. Although finally this pilot study only included 10 patients, given the fact that the population studied corresponds to a very specific subgroup of MM patients, we consider that this study provides new information, although no firm conclusions may be drawn given the number of patients analysed. In contrast, previous studies in MM (the main ones are commented below) have inclusion criteria that are not so strict, and the number of patients included is higher, but then it is very difficult to draw conclusions in these studies for certain subgroups of patients.

Regarding the limitations of our study, another limitation is the lack of similar publications comparing Choline tracers ([^18^F]FCH and [^11^C]FCH) versus [^18^F]FDG PET/CT in the staging of MM. It should be mentioned that a prospective monocentric study, named MYELOCHOL, is under way at the University Hospital in Bordeaux [29], with no results posted yet. Its objective, as in our study, is to compare the detection rate between [^18^F]FDG-PET/CT and [^18^F]FCH-PET in patients recently diagnosed with MM. A group of 30 patients, whose enrolment began in September 2019, will undergo whole-body MRI, [^18^F]-FDG-PET/CT, and [^18^F]FCH PET/CT.

Taking into account the main limitations of our study, future studies should include a larger patient population with a broader spectrum of patients, not only candidates for transplantation. Moreover, it would be interesting to evaluate the lesions that were negative with [^18^F]FCH and evaluate other reference regions different to the liver uptake, because the liver is not an appropriate reference organ due to its high [^18^F]FCH liver uptake. Calabria et al. [30] analysed the physiological distribution of [^18^F]FCH, calculating the mean SUVmax values in various organs in five patients. Interestingly, [^18^F]FCH uptake in the spleen with SUVmax 3.1, a standard deviation of 1.9, and a range of 1.8–4.5 seem to be similar values to those that we usually find in [^18^F]FDG PET/CT in the liver. Therefore, the spleen could be a reference organ for applying Deauville criteria instead of the liver in [^18^F]FCH PET/CT. However, this should be the aim of future research.

Globally, the heterogeneity of the accumulation of different radiotracers seen in the same patient has been described in several studies, suggesting the simultaneous presence of multiple spatially separated clones that coexist in the same patient. In this context, the need to use more than one tracer in some situations which are difficult to interpret should be considered [31]. In fact, the use of multitracer PET/CT is not a novelty in nuclear medicine routine clinical work, as in some tumoral processes, such as neuroendocrine neoplasms, it is widely used, providing information on different metabolic aspects and behaviour [32].

[^11^C]Methionine PET/CT has already been mentioned. It is mainly used in the management of tumours located in the central nervous system [33], and its superiority to [^18^F]FDG PET/CT was first demonstrated in 2013 [34]. Later on, it also evidenced higher sensitivity than choline radiotracers, as published in a head to head comparison study in which [^11^C]-Methionine detected a greater number of intra-medullary lesions in about 40% of patients [22].

As tumoral cells can synthesise membranes by metabolising acetate, [^11^C]Acetate PET/CT has also been studied, demonstrating higher rates of detection than [^18^F]FDG PET/CT [35]. Its utility, as in the case of choline, was initially discovered incidentally [36].

It is worth mentioning the increasing interest in theranostics as nuclear medicine is not only a diagnostic tool, but also has therapeutic clinical applications, with the most clear example being the treatment of prostate cancer [37,38]. The role of [^89^Zr]-Daratumumab [39] and [^90^Y]-labelled pentixather [40] has already been shown in human patients, with promising results in the rates of detection and by offering the possibility of a personalised management of the disease. Despite this, it is still a challenge and too soon to determine which patients would benefit from this new radiotracer. Genetic profiles and other immunogenic parameters may help to select the appropriate radiotracer. The combination of clinicopathological parameters, imaging measurements, and radiomic features is another pathway being used for the development of new prognostic biomarkers [32,41,42].

## 5. Conclusions

Taking into account the main limitation of our study, which is the small cohort of patients included, and given the lack of available evidence on the role of [^18^F]FCH PET/CT in the initial staging of patients with newly diagnosed MM who were candidates for an autologous bone marrow transplant, according to the results of our study, [^18^F]FCH PET/CT seems a promising tool for the initial staging of MM patients. These preliminary results show [^18^F]FCH PET/CT presents a higher detectability of focal lesions than [^18^F]FDG PET/CT, although its role in determining bone marrow infiltration is limited by the physiological bone marrow uptake of [^18^F]FCH.

## Figures and Tables

**Table 1:** Main characteristics of the 10 patients included in the study.

Patient Characteristics							
Patient Number	Age	Sex	Ig	Light Chain	Bence-Jones	% of Plasma Cells	CRAB
1	43	M	IgG	Lambda	No	13	-
2	68	F	IgG	Kappa	No	72.4	Pathological fracture
3	67	F	IgG	Kappa	Yes	17	Anaemia
4	73	M	IgA	Lambda	No	30	-
5	50	F	IgA	Lambda	No	44.6	-
6	59	M	IgA	Lambda	Yes	15	-
7	59	F	IgA	Kappa	No	55	Pathological fracture
8	68	F	IgG	Kappa	No	24.2	-
9	59	F	IgM	Kappa	Yes	25.6	Pathological fracture
10	50	M	IgG	Kappa	Yes	97	Anaemia. Renal failure. Lytic lesions.

CRAB: hypercalcemia, renal failure, anaemia, or bone lesions, known by the acronym CRAB, criteria. Ig: immunoglobulin.

**Table 2:** Description of the location and main characteristics of the MM lesions detected with both PET/CT studies and lesion-by-lesion comparison between [^18^F]FDG PET/CT and [^18^F]FCH PET/CT.

Characteristics of the MM Lesions and Lesion-by-Lesion Comparison between [^18^F]FDG PET/CT and [^18^F]FCH PET/CT												
Patient Number	Bone Marrow Affected		Focal Lesions (Number)		Extra-Medullary/Para-Medullary Disease		Lytic Lesions (Number)		SUVmax in Target Lesion (Location)		Deauville Score	
	[^18^F]FDG	[^18^F]FCH	[^18^F]FDG	[^18^F]FCH	[^18^F]FDG	[^18^F]FCH	[^18^F]FDG	[^18^F]FCH	[^18^F]FDG	[^18^F]FCH	[^18^F]FDG	[^18^F]FCH
1	No	Yes	No	Yes (1)	No	No	No	No	-	4.3 (left iliac)	2	3
2	No	Yes	Yes (2) *	Yes (2)	Vagina and liver foci	No	No	No	-	4.6 (D7)	4	3
3	Yes	Yes	Yes (1) †	Yes (1) †	-	-	Yes (1)	Yes (1)	4.5	4.1	4	3
4	No	Yes	No	No	-	-	Yes (1)	Yes (1)	3.3 (lytic lesion)	2.1 (lytic lesion)	2	2
5	No	Yes (patchy)	No	No	No	No	No	No	-	-	3	3
6	Yes	Yes	No	Yes (3)	Parotid adenopathy	Yes (1)	Yes (1)	Yes (1)	-	7 (D5)	3	3
7	Yes	Yes	Yes (1) #	Yes (7)	Yes (1 PMD)	Yes (1 PMD)	Yes (2) §	Yes (5) §	6.9 (left sacrum)	8.9 (left sacrum)	4	3
8	No	Yes	No	No	-	Preauricular adenopathy	No	No	-	2.7 (adenopathy)	3	3
9	Yes	Yes	Yes (4)	Yes (7)	Yes (1, rib PMD)	Yes (1, rib PMD)	No	No	4.6 (D9)	6.3 (D9)	4	3
10	Yes	yes	Yes (>10)	Yes (>10)	No	No	Yes (>10)	Yes (>10)			5	3

* Both lesions detected corresponded to a rib fracture and arthropathy. † Focal lesion corresponded to plasmacytoma. # The focal lesion detected in patient 7 corresponded to a rib fracture. § All lytic lesions were detected in the cranial calvarium. PMD: Para-medullary disease.

**Table 3:** Description of the SUVmax of the MM lesions detected with both PET/CT studies and lesion-by-lesion comparison between [^18^F]FDG PET/CT and [^18^F]FCH PET/CT.

Description of SUVmax of the MM Lesions and Lesion-by-Lesion Comparison between [^18^F]FDG PET/CT and [^18^F]FCH PET/CT							
**Patient Number**	Axial or Extra-Axial Lesions	[^18^F]FCH			[^18^F]FDG		
		Location	SUVmax	Deauville Score	Location	SUVmax	Deauville Score
1	Axial	Iliac bone	4.3	3	No uptake		
2	Axial	Spinous L5	2.9	3	Spinous L5	2.6	4
		10th right rib	5.3	3	10th right rib	3.0	4
		D6	3.6	3	D6	1.6	1
		D7	4.6	3	D7	1.7	1
3	Axial	Left sacrum	4.1	3	Left sacrum	4.5	4
4	Extra-axial	Internal femoral condyle	2.1	2	Internal femoral condyle	3.3	2
5	Axial	No uptake			No uptake		
6	Axial	D5	7.0	3	No uptake		
		Left sacrum	5.6		No uptake		
		Iliac bone	6.1	3	No uptake		
7	Axial	Sacrum para-medullary lesion	8.9	3	Sacrum para-medullary lesion	6.9	4
		D6	8.0	3	No uptake		
		Calvarium	1.6	3	No uptake		
8	Axial	No uptake			No uptake		
9	Axial	D9	6.3	3	D9	4.6	4
		3rd left rib	4.0	3	3rd left rib	3.5	3
10	Axial and extra-axial	Sternum	5.9	3	Sternum	8.3	5

Axial lesions located in the spine are named as D: dorsal or L: lumbar followed by the affected level.
